# Hospital Meetings

**Published:** 1903-03-14

**Authors:** 


### HOSPITAL MEETINGS.

### THE LONDON HOSPITAL.

The general Court of Governors of the London Hospital held on Wednesday, the 4th inst., was a formal meeting lasting only a quarter of an hour. The Hon. Sydney Holland, who presided, read the report, which was unanimously adopted; and after this the general meeting became special, to sanction certain agreements as to property. The statistics given in the report were as usual very striking: 13,160 in-patients during the year, 162,147 out-patients, 3,499 children under 12, and 1,404 Hebrews as in-patients; 9,934 anæsthetics administered, an average of 30 a day. The average stay was 19·21.

The following particulars as to finance were given by the Chairman:—

The total expenditure on the maintenance and management of the hospital was £87,550, whilst the extraordinary expenditure amounted to £93,239. Of this extraordinary expenditure about £3,000 is a certain expenditure sure to recur every year, and therefore may be considered as ordinary expenditure, making a total of the ordinary expenditure over £90,000. The ordinary income has amounted to £65,700, and in addition there has been a grant from the King Edward's Fund of £8,500, and the legacies which have been received during the year amounted to £23,641. All of these legacies had to be used to meet current expenditure. The London Hospital is making this year a special quinquennial appeal for funds to carry on the work during the ensuing five years. As the total expenditure, ordinary and extraordinary, was £180,790 in 1902, land land investments were sold, and the capital account of the hospital was thus reduced by £77,131. It is estimated that £167,000 more must be spent on the improvement scheme which is being carried out, and for this sum an appeal is made, whilst to place the hospital on a sound financial basis, at least £25,000 per annum increase in ordinary subscriptions and donations is necessary.

Mr. Holland reported that the various alterations and improvements were making good progress; the coroner's court had been arranged for, to be rented from the hospital by the County Council; a much larger clinical laboratory would be provided; the carrying power of the lifts would be increased; and orders had been given to proceed with the alterations in the west wing. In this wing, the chairman added, provision would be made for small separate wards for twenty-four-hour and noisy cases.

Mr. Ernest W. Morris was appointed secretary in place of Mr. G. Q. Roberts, and it was decided to discontinue the office of House Governor, with which the secretariat has hitherto been combined. A list of subscriptions and donations received in response to the quinquennial appeal was laid on the table; the sums amounted to something like £100,000. The chairman announced that the Mayor of Stepney had arranged to hold a public meeting to further the claims of the hospital in the borough; he hoped that other local boroughs would follow suit.

### ROYAL WESTMINSTER OPHTHALMIC HOSPITAL.

Sir Charles Turner, K.C.I.E., who presided at the annual meeting of this hospital, on Friday the 6th inst., said he understood that at a recent meeting of gentlemen connected with hospitals, held at Caxton Hall, Westminster, some observations were made as to the propriety of removing this hospital from its present site. He thought that the question of loss, entailed by the sacrifice of buildings of recent reconstruction, ought to be taken into consideration. The committee, he continued, took counsel before the improvements were begun, and they found that they could not reasonably hope to build a hospital with the accommodation they desired, on a site which would be equally convenient to the public, for less than £70,000. Having kept the subscription list open, and having invited contributions towards the rebuilding on a new site, during five years, barely £4,000 was received. It was, therefore, quite impossible to contemplate removal without incurring blame for extravagance. The committee, further, had made it a rule to cut its coat according to its cloth, and to avoid incurring debt as far as possible, and when they set to work to re-model on the existing site, they were careful to keep the expenses down to the limits of their power. The original estimate for reconstruction was between £5,000 and £6,000, but it was found that something like £9,000 would have to be spent to make the work complete, and the committee decided to do at once all that was necessary. Every shilling had been well spent, but none of the money specially subscribed to the building fund had been drawn upon, except where the givers desired it to be used, and the £3,000 remaining formed the nucleus of a building fund, in case the hospital were obliged hereafter to remove. The sum put into the building fund (£2,635) by the general fund had been withdrawn, in order to meet the additional expense incurred in the improvements, which included the strengthening of the stone staircases with iron supports and girders. Towards this expenditure the council of King Edward's Fund had given the liberal amount of £500; the committee were aware that the hospital was recommended for a very much larger amount, and he trusted that in future years more might be given. £50 had been received from the Dean and Chapter of Westminster, being money paid by visitors to the Abbey after the Coronation. The City Corporation had given £50, the Sunday Fund £154, and the Saturday Fund £62 12s. Many donors encouraged the committee to believe that if they maintained the hospital in the future as they had in the past, every support would be accorded, and the debt paid off it would shortly be reduced to £3,500 by the sale of railway stock. He concluded with a reference to the liberality of the staff during the progress of the improvements; and, speaking of their warm personal interest in the work, he said he believed that if the hospital were removed to the outskirts of London the public would lose a very great deal, and the hospital could not possibly discharge so much charitable work as at present. No other remarks were offered on the subject, and the report and accounts were unanimously adopted.

### MOUNT VERNON HOSPITAL FOR CONSUMPTION.

Sir Henry Burdett, who took the chair at the annual meeting of the Mount Vernon Hospital for Consumption, commented upon the fact that the Royal Hospital for Diseases of the Chest had asked that beds might be reserved for their patients in the Mount Vernon Hospital. This request showed that the authorities of the Royal Chest Hospital recognised that the accommodation at their hospital in the City Road was not up to modern requirements. The sooner this hospital was moved out into the country to a fitting site the better.

Sir Henry quoted a saying of Mr. Gladstone's made in 1863 when there was much discussion as to whether hospitals should continue to be maintained by voluntary efforts or by the State:—"It would be a great evil to dispense with all public control: hospitals want supervision." Latterly public interest in hospitals had been much stimulated, and public control through the governors had been increasingly exercised with excellent results. Great funds had come into being such as the Sunday and Saturday Funds, and more recently the King's Hospital Fund. This last had been of the utmost service, especially by representing the public, visiting the hospitals, and recommending changes where necessary, which had greatly strengthened public confidence in the proper working of the hospitals. This was evinced by a very large increase in the volume of support and a wider area of givers, so that to-day the voluntary principle of hospital support was permanently secured. That this was essential was proved by the fact that under no other system could anything like the same high degree of efficiency be met with, nor the same progressive development in every department of hospital life be secured as existed to-day in our hospitals. Public control was chiefly exercised by the Governors and should be expressed by their regular attendance at the annual meetings, but there was much room for improvement in this particular. It was well worth the while of the secretary and committee to endeavour to attract the Governors to these meetings, for the increase of interest meant an increase of the subscribers. Non-attending Governors lost the right of protest even in case of abuses. Sir Henry then gave a brief sketch of the history of the hospital during the last 40 years, from its first beginning in 1860 until the year 1883, when, through mismanagement, its affairs got into so serious a state that an investigation committee was appointed, for which Sir Henry acted as honorary secretary. As a result of that investigation the hospital was entirely remodelled, and having had the good fortune to secure the services of its present able chairman, Mr. B. A. Lyon, it was launched upon a most successful and prosperous career. It might be said that in these days it was impossible that the old abuses could still exist in hospitals, but the truth was that unless certificates of fitness were required of all new institutions irregularities would still continue. He quoted as an instance a hospital in Soho where a most scandalous state of things existed, whilst one in Brompton and one in Kennington were also unfit for the purposes they were supposed to fulfil.

The most satisfactory feature of the past year was the organisation of a new hospital at Northwood. This had been built by the munificence of an anonymous donor who made the sole stipulation that everything should be done to render it the most efficient sanatorium for the treatment of consumption possible. The hospital at Northwood would be the first completed on the very best basis, and they must be prepared to find it imitated. In conclusion Sir Henry paid a warm eulogy to the excellence of Mr. Lyon's work since he became chairman of the Committee of Management. The hospital needed an additional £600 a year in annual subscriptions, and these should be forthcoming by Easter at latest.

Mr. B. A. Lyon, who was the next speaker, said that the hospital owed a great debt of gratitude to Sir Henry Burdett. They might point with pride to the fact that they had obtained the first grant of £1,000 from the King's Hospital Fund and a further donation of £500. Mr. Lyon then went on to say that extraordinary progress had been made at the Northwood Hospital, and he quite hoped that it would be completed and ready for occupation by the spring of next year.

The usual business having been proceeded with, Lord Clifden moved a vote of thanks to the chairman.

The report for 1902 showed an increase of out-patients from 4,699 to 4,962. There was a diminution noted in both subscriptions and donations, but fortunately the grants received from the Hospital Sunday Fund and Hospital Saturday Fund were larger than in previous years. There was an increase of expenditure which left the hospital with a deficit of £2,469. H.R.H. Princess Christian laid the foundation-stone of the new Country Branch Hospital at Northwood on May 13th of last year.

---

### ROYAL FREE HOSPITAL.

The annual meeting of the Royal Free Hospital, Gray's Inn Road, was held on the 25th ult., the hall being crowded to its utmost capacity. Her Highness, Princess Louise Augusta of Schleswig-Holstein, President of the Ladies' Association, occupied the chair in the absence of her mother, Princess, Christian, who since this time last year has been President of the Hospital.

Mr. Charles Burt, the treasurer and chairman of the weekly board, opened the proceedings by moving that the annual report and financial statement be adopted. The committee were convinced, he said, that the acceptance by her Royal Highness of the position of president had been productive of great benefit to the institution. They had evidence of this in the presence of her Highness her daughter in the chair that day, and that not as an act of form, for he could assure the governors that her Highness took the deepest interest in the work done, and was in fact becoming familiar with the wards, and well-known to the patients there. The report showed that they had had improvements in every department. Ever since last February, when her Royal Highness was appointed, there had been distinct advances in all branches of their work, and he hoped that fact would be conveyed to her. The number of their patients had increased and their finances were in a better condition.

Like other institutions they had gone through hard times, but they had never got into debt, and he hoped they never would. But they lived from hand to mouth, for legacies were a very unreliable source of income. Their revenue from investments was only £1,000 a year; he thought that should be at least £5,000, and they endeavoured always to put aside some portion of their legacies for that purpose. But the best source of income after all was the living gift from the living giver; it blessed both the giver and the receiver, but especially the giver.

### Hopes and Intentions.

They were looking forward to the great bazaar to be held in June which their President and the Ladies' Association hoped to make the success of the season, since money was wanted for many things. They had carried out many improvements and they had many more still in contemplation. The chief of these were the readjustment of the sanitary arrangements of the Sussex wing; they wanted to have the electric light permanently brought in; and the surgeons told him they badly required a new operating theatre. All these things would, of course, absorb a large sum of money and he hoped it would be forthcoming. During the last fourteen years they had spent some £53,000 in rebuilding and adding to the hospital. Every one of these alterations and additions had been a necessary and a desirable one, and tended to the success of the institution and to the comfort of those who lived there, whether as patients or as staff. The fire which in November last broke out in the ironing-room of the laundry showed that their people—both men and women—could keep their heads, with the result that no damage was done except in the place where the fire actually began. There were many other matters which the meeting would excuse him for not dealing with then, but he could not sit down without referring to the appointment of two eminently qualified ladies—Mrs. Scharlieb, M.D., and Miss Ethel Vaughan, M.D.—to act as physician and assistant physician for the diseases of women. The new departure was working well, and he congratulated the ladies of the medical school with which they were so closely connected, and hoped these appointments would be the precursors of similar appointments in other hospitals.

The report having been adopted, Mr. Sheppard proposed that Mr. Justice Bruce be appointed a vice-president of the hospital. It was a matter of regret to them all he said that, owing to the pressure of other engagements, he felt obliged to give up the chairmanship of the committee, and they wished in this way to maintain his official connection with the hospital and to recognise his great services in the past. This was carried, and the committee and officers re-elected, after which Mr. Layland Barratt moved a vote of thanks to the Ladies' Association, started in May last. Considering the work which the hospital had done for women, he said it was only fitting that women should take an active interest in and work for the interests of the hospital, and this was now being done.

Replying to the vote of thanks to the medical staff, Mr. Barrow, the senior surgeon, emphasised the need for a new operating theatre, declaring that theirs was now the worst in all London, a sentiment in which Mrs. Scharlieb agreed, it being difficult, she said, for her and her students to get in at all so cramped for space and time were they. Mr. Burt held out the hope that the committee would shortly see their way to erecting a new operating theatre at the top of the building, and said they were quite ready to receive subscriptions for the purpose.

Praise from Sir Edmund.

Sir Edmund Hay Currie expressed warm approval of the work of the matron and nursing staff, and went on to say that he thought the Secretary's (Mr. Conrad Thies) system of accounts was as perfect as could be. Doubtless it was due in some measure to this that that hospital had not increased its maintenance cost of 28s. per week which was what it stood at ten years ago. It was distinctly to the credit of the Royal Free that it had been able to hold its own and carry on at a cost per bed which was the lowest of any hospital with a medical school attached. If they had spent the £2 a week which was spent in most hospitals they would not be in the satisfactory financial position they were.

Mr. Thies in reply modestly gave the credit for so desirable a state of things to the weekly Board. If, said he, Boards generally would adopt the same system of careful watchful considerate control of every item of expenditure, there would be less heard of complaints of increased cost of maintenance.

The Princess having expressed the pleasure it gave her to be present at the meeting and to do anything in her power for the good of the hospital, the proceedings terminated, after which visits of inspection were made to the various wards.

### ROYAL ORTHOPÆDIC HOSPITAL.

Plans for the New Building Approved by the Governors.

No discordant note was sounded at the annual meeting of the Royal Orthopædic Hospital, held on the 26th ultimo. The report and accounts were adopted without question and no opposition was offered to any of the names proposed for the committee of management, to which two ladies, the Countess of Denbigh and Mrs. James Annan were elected.

Mr. Ernest Flower, M.P. (the deputy chaiman), presided, and in moving the adoption of the report and accounts for 1902 expressed regret at the absence of Mr. Harry Marks (the chairman of the hospital), who was detained at home by a severe illness, which he was sure all those present hoped would be of brief duration and that they should soon again have the benefit of his kindly counsel and assistance. There were not many points, he said, on which it was necessary to make any comment. It was a regrettable fact that they were in debt to tradespeople and others to the extent of close on £2,000. This had not been occasioned by the ordinary work of the maintenance of the hospital but by sanitary work which the committee had to embark upon two or three years ago, under pressure from the health authorities. This had hung round their neck like a millstone, but the prospect was now not without comfort. The expenditure last year showed a substantial reduction, which was due not only to the closing of some of the beds but to more economical and efficient management. Beyond that it had come to their knowledge during the last few days that the hospital was to receive a legacy of a substantial amount, and if their friends would help them he hoped that when next they met they would have wiped off that debt altogether. It was most desirable that it should be so, because what was uppermost in their minds was the removal of the institution to the new premises in Coram Street, the plans for which were before them that day. They hoped to leave the old premises free from debt, and in making their appeal on behalf of the new building fund to make it *ad hoc* and not to pay off old debts. Last year they were in some doubt if they would be able to obtain a lease of 99 years, but this had now been granted, and he wished to pay a tribute to the kindness and consideration with which the Duke of Bedford and his agents had treated them in the matter. The committee had entered into a preliminary contract for the erection of the first part of the building, and this was now being proceeded with by the builders. They had, therefore, to face some considerable expenditure from day to day, and they proposed to issue very shortly an appeal to the public for about £25,000. Mr. Marks had already promised £500, Mr. Kauffman £250, Mr. Thomas and another friend £100 each. King Edward's Fund voted them £250 some years ago for this purpose, and altogether they had about £2,050 already subscribed.

The Question of Special Hospitals.

There was a matter which had lately been ventilated in the newspapers, that of the suggested amalgamation of the special hospitals. He had been approached by one of the secretaries of King Edward's Fund with a view to find out what would be their attitude toward the question. There were now three orthopædic hospitals in London, each doing exceedingly useful work; and though of course they were friendly rivals in the matter of subscriptions, yet they were all working together for the relief of the poor and suffering. They were prepared to consider in the most sympathetic way any proposals for the amalgamation of the three kindred institutions, and he might point out that in the steps they were taking in the removal from their present site and the creation of a model hospital on their new site they were not doing anything contrary to such a step but what would be of help if it were decided upon. One of the objects of the proposition was that there should be provided a central hospital where patients could be treated with the best skill available, while allied to it there would be branches in more healthy and outlying neighbourhoods for patients not requiring such continuous treatment by specialist skill so much as proper nursing, appliances and treatment under skilled supervision and observation. He certainly should not like it to be thought that the Royal Orthopædic would desire to do anything but co-operate heartily with others in such a movement. One other matter he must mention, and that was the great obligation they were under to their honorary officers during the past 12 months. Their honorary architect, Mr. Walter Emden, had with unfailing patience prepared plan after plan, until at last had been evolved what he thought might fairly be called a perfect orthopædic hospital. On their honorary solicitors, too, had fallen a great deal of work during the year.

Mr. Biddulph Martin, M.P., the treasurer, said he looked on the removal as a regrettable step, but it had been found impossible to maintain the hospital in efficiency on its present site. He thought they were fortunate in obtaining a new site on such favourable terms as they had done, and felt sure the change would be conducive to good work. As to the question of amalgamation there was much to be said in its favour, and he also thought the question of affiliation with one of the great general hospitals might be considered. In the country they could have branches on cheaper land and with purer air, while in London they had the advantage of skilled operative surgeons.

The election of the committee was then proceeded with and votes of thanks brought the proceedings to a close.

### THE CANCER HOSPITAL.

There was a goodly attendance at the annual meeting of this hospital on Wednesday (25th ult.), when Mr. William Hooper was voted to the chair.

The Chairman said that but for one thing, the past year had been one of prosperity; the death, however, of Dr. Alexander Marsden had thrown a gloom over the report. He had been connected with the hospital since its foundation, and its success was in no small measure due to him.

Statistics showed that cancer was increasing, and in this hospital 732 in-patients and 1,709 out-patients were treated in 1902, an increase of 28 and 105 respectively. With regard to finance, £13,000 a year was required to keep the hospital going. Although there was an increase of £90, the annual subscriptions were only £2,200 last year, and the committee were very grateful to King Edward's Fund for the grant of £200, as well as to the Sunday and Saturday Funds for £524 and £185 respectively. The first, in making its grant, again referred to the hospital as "an excellent institution." Cancer Research had recently been stimulated by the provision in the hospital of a new laboratory. The committee had thus added considerably to their burden, but they felt it was the wish of the governors that everything possible should be done to solve the mysteries surrounding this terrible disease. Wherever else the cause of cancer might be discovered, this hospital must take its share, and he hoped the discovery would be made within its walls.

Dr. Snow said he hoped that the simple preliminary step of defining terms would be taken by the committee; much confusion existed in the medical world with regard to the various forms of cancer, and most of the figures collected were almost utterly useless on this account; people working at cancer investigation ought to know exactly what they were working at. A preliminary conference ought to be held to determine on what lines investigation should proceed.

### HOSPITAL FOR CONSUMPTION, BROMPTON.

Mr. H. C. Deedes presided over the Quarterly Court of this hospital on Thursday (26th ult.). It was announced that the building of the country branch and convalescent home for open-air treatment at Heatherside, near Frimley, had made satisfactory progress, but the committee regretted that the subscriptions for this special work were coming in very slowly. The award of £1,000 from King Edward's Hospital Fund was most acceptable.

